# Editorial: Effects of pharmacologic therapy for diabetes mellitus on the endocrine system

**DOI:** 10.3389/fendo.2022.960930

**Published:** 2022-07-14

**Authors:** VA Giagulli, G Reimondo, P Moghetti

**Affiliations:** ^1^ Interdisciplinary Department of Medicine-Section of Internal Medicine, Geriatrics, Endocrinology and Rare Diseases, University of Bari "Aldo Moro", School of Medicine, Bari, Italy; ^2^ Santa Maria Hospital, Gruppo Villa Maria (GVM) Care & Research, Bari, Italy; ^3^ Internal Medicine, Department of Clinical and Biological Sciences, San Luigi Gonzaga Hospital, University of Turin, Turin, Italy; ^4^ Endocrinology, Diabetes and Metabolism, Department of Medicine, University and Hospital Trust of Verona, Verona, Italy

**Keywords:** diabetes mellitus, sodium-glucose cotransporter-2 inhibitors, glucagon-like peptide-1 receptor agonists, aldosterone, testosterone, parathyroid hormone, thyroid hormones, growth hormone

Among non-communicable diseases, type 2 diabetes mellitus (T2DM) is significantly increasing not only in the western populations but also in those from East and South Asian countries such as China and India. Therefore, its macrovascular (i.e. cardiovascular) and microvascular damages are expected to rise worldwide, with a significant increase in social and economic burden of diabetes (1).

In those diabetic patients not achieving therapeutic targets by means of lifestyle measures, pharmacologic therapy must be considered. Interestingly, the new glucose-lowering agents - in particular glucagon-like peptide 1 receptor agonists (GLP-1 RA) and sodium-glucose cotransporter 2 inhibitors (SGLT2-i) - have been associated with a number of extra-glycaemic effects, including cardiovascular benefits, nephron protection, and weight loss, reducing both morbidity and mortality in patients with type 2 diabetes and substantially improving the clinical management of this condition (2).

Indeed, there is growing evidence that the decision to treat higher-risk subjects with GLP-1 RA or SGLT2i to bring down rates of major adverse cardiovascular events (MACE), cardiovascular disease (CVD), hospitalization for heart failure (CHF), cardiovascular death, chronic kidney disease (CKD) progression, or even cognitive decline, can be taken regardless of baseline HbA1c or specific HbA1c values. Moreover, GLP-1 RA can also be used in nondiabetic subjects with obesity, while SGLT2i are recommended in patients with or without T2DM and heart failure, with and without reduced ejection fraction, to reduce CFH, MACE, and CVD death. Moreover, they can be now prescribed in patients with or without type 2 diabetes with CKD to prevent the progression of this complication and even cardiovascular death (3). Additionally, SGLT2-i appears to improve all heart outcomes independently of the frailty status in individuals with HF and a left ventricular fraction of 40% or less and with or without T2DM (4). Finally, GLP-1 RA appear to positively affect both the small vessels and endothelial function, as proved by the enhancement of erectile function in diabetic men with previous cardiovascular events, and even in those with obesity and overt hypogonadism (5, 6).

The therapeutic efficacy of both these glucose-lowering drug classes on metabolic control appears to be due to both direct and indirect actions on several tissues, such as the central nervous system, adipose tissue, and liver. There is evidence that GLP1-RA can integrate a network of neuronal and endocrine signals and may achieve this by a number of potential mechanisms, including regulation of gastrointestinal transit, stimulation of insulin secretion, activation of satiety pathways in the central nervous system, and reduction of hepatic glucose production, ectopic lipid accumulation, and inflammation (7). Similarly, SGLT2-i appear to restore hypothalamic insulin sensitivity, with potential favourable effects on glucose levels and liver fat accumulation. Some of the beneficial effects of this class of anti-hyperglycaemic substances, on different organs of the body, may rely on brain-periphery cross talk and hormones *via* the parasympathetic nervous system (8). However, only limited evidence is still available on the effects of this pharmacologic therapy on neuronal and endocrine systems. Indeed, further studies are needed to fully understand the mechanisms underlying the effects of those glycaemic-lowering compounds on metabolic control, diabetic complications and other abnormalities frequently occurring in diabetic patients (e.g. osteoporosis, male hypogonadism, hyperandrogenism in women, hypertension, NAFLD, etc.). Among these aspects, of great interest is to understand the potential effects of these medications on other endocrine systems.

In their review, Cignarelli et al. explored the possible involvement of the GH/IGF axis in the pleiotropic effects of GLP-1RA and SGLT2i compounds. In particular, the possible anti-apoptosis action of GLP-1 RA on β‐cells, heart, brain, and bone might potentially be modulated by the IGF-1/IGF-1R system, given its crucial role in those organs. Moreover, SGLT2i improve IGF-1 expression in muscle mass, hindering its loss and promoting contractile force recovery in a mouse model of obesity. The IGF-1/IGF-1R system might also participate in facilitating hepatic ketogenesis, a phenomenon, which was reported in diabetic subjects treated with SGLT2i. However, the authors underline that very little information, especially based on preclinical data, has been reported in the literature thus far, still precluding definite conclusions on the role of the GH/IGF axis in the pleiotropic effects of GLP-1RA and SGLT-2is compounds.

Both T2DM and primary hyperparathyroidism (PHPT) can negatively affect bone health, facilitating bone mass loss and increasing the risk of fracture. Based on this background, Castellano et al. reviewed the relationship between T2DM and its treatment and bone manifestations of PHPT. Although limited evidence are available, they retrospectively examined a large consecutive series of 472 subjects with PHPT. Among them, 55 patients suffered from both PHPT and T2DM. These subjects were older, mildly symptomatic, and with a lower prevalence of radiological bone features, as compared with PHPT subjects without T2DM. Unfortunately, this cohort included very few subjects who were given the new classes of antidiabetic drugs, and the potential effects of these compounds could not be explored.

The mechanisms underpinning the cardio-nephro-protection exerted by both abovementioned classes of antidiabetic agents remain only partially understood. The descriptive review from Puglisi et al. investigated the relationship between SGLT2-i and GLP 1-RA and the renin-angiotensin-aldosterone system (RAAS), which appears to be activated in individuals with T2DM. Interestingly, in the initial treatment of T2DM patients, SGLT2-i brings about a plasma renin activity (PRA) increase. However, PRA and aldosterone seems to be unaffected in chronically treated patients. Additionally, recent studies have provided evidence that SGLT2-i might have an interfering effect on the aldosterone/renin ratio in patients with T2DM, likely due to their diuretic and sympatho-inhibitory effects. Conversely, the favourable effect of GLP-1-RA in terms of cardio and neuroprotection might at least partly be due to their interaction with RAAS: i) they inhibit the angiotensin II synthesis and activity, by deactivating its circulating form and reducing its action on target tissues such as glomerular endothelial cells and cardiomyocytes; ii) they stimulate natriuresis through inhibition of Na+/H+ exchanger 3. However, further studies are required to fully understand the impact of these phenomena on relevant clinical issues.

Non-alcoholic fatty liver disease (NAFLD) can progress towards non-alcoholic liver steatohepatitis (NASH), cirrhosis or even hepatocellular carcinoma. These alterations in the liver can coexist with T2DM or even precede the onset of T2DM. Therefore, treatment of patients with T2DM may require an accurate choice of medications, with the goal of improving both dysglycaemia and hepatic damage. GLP-1 RA reduce fat deposits in the liver in patients with NAFLD/NASH (7). Similarly, several clinical trials showed that SGLT2-i could reduce fat content in the liver, being potentially useful for treating NAFLD/NASH and T2DM. Yabiku’s review addresses these clinical aspects, trying to identify the mechanisms by which SGLT2-i may reduce liver fat accumulation.

Several dysmetabolic conditions, including obesity, metabolic syndrome and diabetes mellitus, can damage the male reproductive system, often causing a reduction of serum testosterone (T) levels, leading to overt hypogonadism. Indeed, more than 30% of adult T2DM men have serum T levels < 300 ng/dl which is the cut-off for diagnosing hypogonadism in adults, as defined by the most important international societies in the field (9). The pathophysiology of this hormonal dysregulation is complex, likely including the effects of different mechanisms, such as glycaemic and lipid control, insulin resistance, and sleep apnoea. However, the main role appears to belong to abdominal obesity and to the associated inflammatory state, which damages the hypothalamus–pituitary–gonadal (HPG) axis, as extensively reviewed by Pelusi’s manuscript. Since growing evidence suggests that men with low serum T have higher all-cause mortality rates than men with normal/higher testosterone levels, it would be important to assess serum T in adult diabetic men and to understand the possible effects of glycaemic-lowering compounds in this phenomenon. In a recent prospective real-life observational study from Cai et al., 80 men with T2DM treated with premixed insulin were subdivided into 2 groups based on whether they were given metformin in addition to insulin. These authors found that pre-treatment testosterone levels were similar in the two groups. However, 3-month metformin therapy (plus insulin) was associated to reduced T levels, as compared to patients not receiving metformin, independent of improvement of glucose control. If this preliminary evidence would be confirmed, T levels should be monitored when using metformin in diabetic men. Data in the literature regarding dipeptidyl peptidase-4 inhibitors (DPP4-i) and SGLT2-i treatment for T2DM men with hypogonadism have been scarce and conflicting. Conversely, clinical studies in men with T2DM and hypogonadism receiving GLP1-RA produced promising results, as Pelusi work reports. However, available information is insufficient and further research is needed.

This still limited body of work indicates a potential great interest in the extra-glycaemic actions of these new classes of antidiabetic drugs, particularly on the endocrine system. It can be expected that in the next years the choice of the pharmacological treatments for subjects with type 2 diabetes will be increasingly done not only according to HbA1C targets, costs, and potential side effects, but also for the capacity of drugs to prevent and/or treat organ damage, including in this concept both classical and non-classical targets (i.e. kidney, heart, bone, testis, etc.). Nevertheless, further studies are required to clarify the relationships between antidiabetic drugs action and effects on these targets, independently of glucose control.

## Author contributions

All authors listed have made a substantial, direct, and intellectual contribution to the work and approved it for publication.

## Conflict of interest

The authors declare that the research was conducted in the absence of any commercial or financial relationships that could be construed as a potential conflict of interest.

## Publisher’s note

All claims expressed in this article are solely those of the authors and do not necessarily represent those of their affiliated organizations, or those of the publisher, the editors and the reviewers. Any product that may be evaluated in this article, or claim that may be made by its manufacturer, is not guaranteed or endorsed by the publisher.
